# Correcting serum phosphate homeostasis does not alleviate the health dangers of high phosphate consumption

**DOI:** 10.1186/s12967-026-08624-x

**Published:** 2026-07-22

**Authors:** Jamie L. Arnst, Uma D. Alappan, Manjula Viggeswarapu, George R. Beck

**Affiliations:** 1 The Atlanta Department of Veterans Affairs Medical Center, 1670 Clairmont Rd, Decatur, GA 30033 USA; 2 Department of Medicine, Division of Endocrinology, Emory University, 101 Woodruff Circle, 1026 WMRB, Atlanta, GA 30322 USA; 3 The Winship Cancer Institute, Emory University School of Medicine, Atlanta, GA 30322 USA

**Keywords:** Dietary phosphate additives, Bone metabolism, FGF23, Lanthanum Carbonate, Vitamin D, Calcium, Cinacalcet

## Abstract

**Background:**

It is widely recognized that dysregulated mineral balance, in the context of disease, has negative health consequences. However, growing evidence shows that imbalances in mineral homeostasis from a sustained high dietary intake of inorganic phosphate/phosphorus (Pi), even when kidney function remains clinically normal, can also cause serious health problems, including bone disease. Western-style diets, which commonly include added Pi salts, contribute significantly to the issue. Despite this, investigations into therapeutic strategies to counter these effects in the absences of disease are limited. This study aimed to determine whether clinically used pharmacologic and nutritional interventions could regulate mineral homeostasis and prevent bone loss while correcting the systemic consequences of chronic high dietary Pi intake.

**Methods:**

Healthy mice were fed diets with high (HPD), normal (NPD), or low (LPD) levels of Pi additives and constant calcium (Ca). Additional groups were treated with HPD and supplemented with Vitamin D, Lanthanum Carbonate, Cinacalcet, Zoledronic acid, or a strategy of balancing dietary Ca:Pi.

**Results:**

The therapies that partially or fully corrected serum Pi levels and homeostatic factors, interestingly, corrected the increase in cortical porosity but did not correct the HPD-induced decrease in cortical or trabecular bone volume. Only Zoledronic acid successfully restored the bone volume lost due to HPD but exacerbated the HPD increase in cortical porosity. Surprisingly, many of the treatments generated an inflammatory response in kidney in the context of HPD, suggesting that they can have harmful side effects in the absence of overt kidney disease.

**Conclusions:**

This work advances the understanding of the health impacts of Pi additives which are common in ultra-processed foods and common in the Western style diet. Specifically, the results produce clinically relevant findings that simply controlling serum Pi and/or Ca levels or Pi responsive endocrine factors such as FGF23, PTH, and osteopontin are not sufficient to block the negative impacts of chronic high dietary Pi consumption on the skeleton. Further, therapeutic strategies to control serum Pi have an impact on the kidneys in the context of high dietary Pi intake.

**Supplementary Information:**

The online version contains supplementary material available at https://doi.org/10.1186/s12967-026-08624-x.

## Background

Disorders of phosphorus/inorganic phosphate (Pi) homeostasis, leading to hyperphosphatemia, can arise from kidney disease, inherited syndromes contributing to primary and secondary hypo- or hyperparathyroidism [1], or genetic mutations of Pi systemic regulatory genes [2]. Sustained hyperphosphatemia contributes to abnormalities in bone and mineral metabolism, cardiovascular and kidney disease [3], and in the context of chronic kidney disease (CKD), an increased risk of mortality [4, 5]. Emerging evidence suggests that diets high in Pi-additive salts, as found in ultra-processed foods (UPFs) for example, can produce similar adverse effects, even in individuals with normal renal function. UPFs are high in sugars, inorganic salts, artificial flavors, colors, and preservatives [6]. With UPFs showing negative impacts on the skeleton [7] and increased cardiovascular disease risk [8]. UPFs are often high in Pi salts [9, 10], which are used to enhance taste, texture and shelf life [9]. Diets high in Pi salts can elevate serum Pi levels for up to 8 hours post-consumption [11, 12]. Many Americans consume three to four times the FDA and IOM recommended Pi levels due to the prevalence of UPFs in the Western diet [13]. Even with normal kidney function, this intake can lead to transient hyperphosphatemia [10].

Importantly, chronic high Pi intake, has been linked to adverse health consequences, such as cancer, albuminuria, dementia, renal, cardiovascular, and bone disease [13–30]. Despite this, the underlying mechanisms are poorly understood, and research is limited on how modulation of Pi homeostasis can impact health. Further, age may also play a role in the physiological response. Although in healthy individuals dietary Pi intake, absorption, and serum levels generally decline during aging [31, 32], the aging process results in a general decline in tissue responsiveness to changes in the extracellular environment leading to decreased elasticity and increased senescence and ectopic calcification [33, 34]. High dietary Pi intake is suggested to exacerbate these conditions [10, 35–37] and therefore may generate more profound negative health consequences with aging.

Imbalances of serum Pi or Ca can be influenced by dietary interventions. In the absence of disease, the influence of dietary consumption of Pi or Ca on serum levels can vary depending on context. In the context of adequate intake, excess dietary Pi will still strongly influence serum levels of Pi, however increased dietary Ca produces a minimal change in serum levels. In the context of inadequate levels of Pi or Ca, dietary consumption of either mineral can influence serum levels, although the degree of change with Ca being more limited than Pi. In addition to diet, both minerals are also regulated by circulating factors. Therefore, in healthy individuals, serum Pi levels reflect both dietary intake and the kinetics of kidney excretion, regulated by increases in circulating Pi-responsive endocrine factors [38–40] (phosphatonins) such as Fibroblast Growth Factor 23 (FGF23), Parathyroid Hormone (PTH), the cytokine-like circulating factor osteopontin (OPN), and decreases in vitamin D (VD) [41, 42]. These factors act primarily on the skeleton, kidneys, and intestine to regulate Pi homeostasis by increased excretion or retention and modulating calcium (Ca) levels, in part by changes in deposition or release from bone. The coordinated regulation of these two minerals is important because at atypical and elevated concentrations they can form non-functional precipitates that have negative health consequences [43]. How these factors interact with changes in serum mineral, metabolites, and cell responses to generate tissue dysfunction remain unclear.

The kidney plays a central role in Pi homeostasis. FGF23 and PTH reduce Pi reabsorption by inducing the internalization of Pi transporters (Slc34a1 and Slc34a3) in the proximal tubule, with FGF23 requiring the FGF receptor and co-factor α-Klotho and PTH signaling through the G protein–coupled PTH receptor (PTHr1) [40, 44–46]. VD regulates renal gene expression through binding the vitamin D receptor (VDR). Further, circulating VD levels are regulated by renal enzymes 1-α hydroxylase (Cyp27b1) and 24-hydroxylase (Cyp24a1) to make and remove active VD (1,25-dihydroxyvitamin D3 or calcitriol) and 25-hydroxyvitamin D (calcidiol). These signaling networks not only strive to preserve mineral balance but also impact skeletal, cardiovascular, renal, and immune systems, although their broader roles in this context are still being investigated.

Due to the negative health consequences of dysregulated Pi homeostasis in CKD and secondary hyperparathyroidism, pharmacological strategies for managing hyperphosphatemia or changes in associated Pi-responsive endocrine factors have been developed, including: 1) Lanthanum Carbonate (LaC) to reduce Pi absorption in the gut, 2) Cinacalcet (CNC) (Sensipar) to lower circulating PTH, 3) Vitamin D (VD) to enhance Ca absorption in the gut, and 4) Zoledronic acid (Zol) (Zometa, Reclast) to inhibit bone metabolism and the subsequent exchange of Ca and Pi from bone, the main reservoir of both minerals in the body. Nutritional approaches include: 1) increasing dietary Ca to offset high Pi intake and 2) reducing overall dietary Pi intake. While these strategies show partial efficacy under pathological states, their ability to mitigate the negative effects of chronic Pi additive consumption in healthy individuals, including the negative impacts on bone, remains underexplored.

This study aimed to: 1) evaluate the therapeutic value of using established pharmacologic and dietary interventions potential to protect bone and kidney health during sustained high Pi intake, and 2) dissect regulatory mechanisms of Pi homeostasis using a combination of clinically relevant Pi-network modulators. This study was performed in healthy young mice, using purified diets with varying Pi:Ca ratios, 1:3 (LPD.0.2% Pi), 1:1 (NPD, 0.6% Pi), and 3:1 (HPD, 1.8% Pi), reflecting intake for the highest and lowest 5–10th percentile reported in NHANES data [47–49]. Healthy mice received diets for 5wks or10wks of LPD, NPD or HPD. Intervention groups received HPD supplemented with VD, LaC, CNC, Zol, or a balanced high or low Ca:Pi diet. The primary endpoint was bone volume, trabecular, cortical, and cortical porosity. Secondary endpoints included serum minerals, Pi-responsive endocrine factors (FGF23, PTH, OPN, VD), and kidney gene expression. Interventions that corrected specific HPD-induced changes in serum or gene expression generally corrected the HPD-driven increase in cortical porosity while only Zol, an osteoporosis therapy, prevented the HPD-associated decrease in trabecular and cortical bone volume. Collectively, results identify that existing therapeutic strategies to normalize serum Pi or Pi-responsive endocrine factors show limited efficacy in correcting high Pi additive driven reduction in bone volume, highlighting the need for new approaches to prevent tissue dysfunction driven by excessive dietary Pi intake. This study advances our understanding of Pi regulation and its systemic consequences, providing a foundation for translational strategies to mitigate long-term health risks of high dietary Pi.

## Methods

### Study design and diets

Female or Male C57BL/6J mice were obtained at 8wks of age from The Jackson Laboratories (Bar Harbor, ME) and housed in a facility with controlled conditions (temperature, 21–24 °C; humidity, 40–70%; light/dark cycle, 12/12 h) and had *ad libitum* access to diets and water. At 10wks of age, mice were randomly assigned to varying Pi diets (fed *ad lib*) synthesized by Inotiv/Envigo (Teklad) (Madison, WI), consisting of a normal-phosphorus diet (NPD; 0.6% phosphorus [TD.140507]), low-phosphorus diet (LPD; 0.2% phosphorus [TD.110360]), or high-phosphorus diet (HPD; 1.8% phosphorus [TD.110362]). These casein-based purified diets are designed to be isocaloric (3.7–3.9 kcal/g) and keep calcium (0.6%), vitamin D (2.2 IU), and other vitamin and minerals constant. Three additional HP diets were used with compounds incorporated during diet synthesis: i) HPD with Vitamin D (cholecalciferol; added by Inotiv, Madison, WI) (HPD+VD) added at 10 IU/g diet, ii) [TD.190548], ii) HPD with Lanthanum Carbonate (Sigma-Aldrich: #325767) (HPD+LaC) at 3% [TD. 190549] and iii) HPD with Cinacalcet (HPD+CNC) (InvivoChem, #V1491) with 100 mg/kg [TD.190549]. A fourth group was fed HPD and injected intraperitoneal with Zoledronic acid (Selleck Chemicals, #S1314) (HPD+Zol) 100 µg/kg once at the start of the study. The low Ca-low Pi diet (LCPD) was the same diet as the LPD with Ca lowered to 0.2% [TD.140831] and the high Ca-high Pi diet (HCPD) based on HPD with Ca raised to 1.8% [TD. 140832]. The institutional chow “house” was (Global 18% Protein Rodent Diet [2018]; Inotiv/Envigo) a grain-based diet with 0.7% Pi (includes ~0.3% phytate) and 1.0% Ca. Key aspects of the experimental diet compositions are shown in Supplemental Table 1. Body weights were recorded on day of sacrifice. No adverse events were noted. Animal studies were approved by the Institutional Animal Care and Use Committee of the Atlanta Veterans Affairs Medical Center and comply with NIH guidelines. Certain data have been previously reported associated with the male mice LCPD, LPD, NPD, HPD, and HCPD [28] and female LPD, NPD, HPD female 10 wk diet mice [20].

### Measurement of fecal Pi and Ca

Pi and Ca were measured in dried feces obtained from cage bedding at sacrifice. For each pellet 2-5 mg was dissolved in 5% nitric acid ICP-OES (Thermo Fisher Scientific, Waltham MA; #T00309) for at least 48hrs and Inductively Coupled Plasma Mass Spectrometry (ICP-MS) performed using a Perkin Elmer NexION 2000. The standards are made by dilution of Spexertificate standards Instrument Calibration Standard 2 (Catalog # CL-CAL-2) and Multi-element solution 4 (CLMS-4) (Thermo Fisher).

### Micro-computed tomography imaging of the femur, lumbar spine, and kidney

Micro-Computed tomography (µCT) was performed on the 3rd lumbar (L3) vertebrae and femur ex vivo to assess trabecular and cortical volume and architecture. The 3rd lumbar vertebrae (L3) and femoral midshaft were imaged and analyzed by Scanco μCT-40 (Scanco Medical, Bassersdorf, Switzerland) following dissection and fixation in 75% ethanol. Images for quantification were acquired at 70kVp, 114 mA, 200 ms integration time, and 6 µm voxel size. Reconstructed images were analyzed using the auto-contour function for vertebral trabecular bone for approximately 350 tomographic slices using a threshold of 220 mg HA/cm^3^, sigma 0.8, and support 1. Cortical bone was quantified at the femoral mid-diaphysis for 99 tomographic slices. The periosteal and endosteal masks were created as previously described [50] and then segmented using a threshold of 800 mg HA/cm^3^, sigma 0.3, and support 1. Cortical and trabecular volumetric and architectural parameters were calculated using standard methods [51–53]. Indices and units were standardized according to published guidelines [52, 54]. For cortical porosity, images of the same femurs as above were acquired by Scanco μCT-50 (Scanco Medical, Bassersdorf, Switzerland) at 70 kVp, 200 μA, 1000 projections, 500 ms integration time, and a 5.0 μm voxel size. Cortical porosity was assessed using a threshold of 600 mg HA/cm^3^, sigma 0.3, and support 1. Images were generated from reconstructed and analyzed objects. For measurement of kidney mineralization, kidneys were dissected and fixed in 10% neutral buffered formalin before imaging on µCT-45 at 70 kVp, 114 µA, 1000 projections, a 400 ms integration time, and 7.5 µm voxel size in air. Calcification for the whole kidney was assessed using standard bone volume (Object Volume, OV) methods and segmented from soft tissue using a threshold of 1000 mg HA/cm^3^, sigma 0.8, and support 1.

### Biochemical indices of serum factors

Serum was collected at the completion of each time point under non-fasting conditions and generally between 9:00AM and 12:30PM. Serum chemistry for Pi and Ca was measured on Abaxis VETSCAN VS2 Chemistry Analyzer (Zoetis, Parsipanny NJ). For pooled samples, equal amounts of serum (10 µl) were pooled from each mouse/group to yield 100 µl. Serum markers of bone metabolism and Pi responsive endocrine factors were quantified using enzyme-linked immunosorbent assays (ELISAs). All ELISA assays were performed according to manufacturer’s protocols and quantified by VERSAmax microplate reader (Molecular Devices, San Jose CA). ELISA kits were purchased as follows: Osteocalcin (60-1305), Fibroblast growth factor 23 (FGF23 C-term; 60-6300 and intact; 60-6800) and Parathyroid hormone (PTH1-84; 60-2305) (Quidel, San Diego, CA), Carboxy-terminal telopeptide of type I collagen (CTX, RatLaps; AC-06F1) and N-terminal propeptide of type 1 collagen (P1NP; AC-33F1) (Immunodiagnostic Systems, Gaithersburg, MD), and osteopontin (OPN; MOST00) (R&D Systems, Minneapolis, MN). Vitamin D 25-OH was measured by ELISA (Eagle Biosciences, Amherst NH, VID21-K01), which utilizes a monoclonal antibody that binds to both 25-OH Vitamin D2 and 25-OH Vitamin D3 equally. For balanced Ca-Pi studies pooled serum was measured for 25(OH)D by IDS iSYS ELISA.

### RNA extraction, cDNA synthesis, and qRT-PCR

RNA from tissue in Trizol (Thermo Fisher) was processed with a Bullet blender and Rhino tubes (Next Advance, Inc. Troy, NY) and RNA from tissue and cell culture extracted using TRIzol reagent following the manufacturer’s protocol. cDNA was synthesized using Geneamp RNAPCR kit (Applied Biosystems, Waltham, MA), and qRT-PCR was performed using EvaGreen qPCR master mix (Biotium, Hayward, CA) on an BioRAD Icycler (Hercules, CA). Primers were designed using qPrimerDepot software [55] or PrimerBank [56] as described previously [20] and detailed in Supplemental Table 2. Fold change was calculated using the 2^(-ΔΔCt)^ method [57] and normalized to 18S.

### Histology

The left kidney was fixed using 10% Neutral Buffered Formalin (NBF) and embedded in paraffin. Sections were dewaxed with xylene and rehydrated with ethanol (100%-50%). Antigen retrieval was performed using a steamer in citrate buffer (10 mM sodium citrate, 0.05% Tween-20, pH 6.0) for 10 min at high pressure. Sections were blocked using 5% normal goat serum and 2% BSA in staining buffer (130 μM NaCl, 7 mM Na2HPO4, 3.5 mM NaH2PO4, 0.02% Triton-X, 0.01% Tween-20) for 1 hr, then incubated with primary antibodies overnight at 4 °C in a humidified box. Primary antibody for Klotho (MAB1819, R&D Systems, Minneapolis, MN) was visualized used ImmPACT DAB peroxidase substrate kit (Vector Laboratories, Newark, CA).

### Statistical analysis

Results are expressed as mean ± SD. Individual mice are graphed as individual values and pooled samples run in triplicate are graphed as solid bars. Base Pi diets (LPD, NPD, HPD) were compared by one-way ANOVA with multiple comparisons test for linear trend between column mean and LPD-NPD-HPD column order. Individual groups were assessed by unpaired two-tailed student’s t test as indicated. The presence of outliers was identified by ROUT (Q = 1%). The data were analyzed using PRISM version 10 (GraphPad Software, Inc., San Diego, CA). All statistical tests were performed at the 5% significance level or more stringent as indicated. Statistically, using pooled samples run in technical replicates is less stringent than biological replicates and data should be interpreted accordingly.

### Study approval

Animal studies were approved by the Institutional Animal Care and Use Committee of the Atlanta US Department of Veterans Affairs Medical Center and comply with NIH guidelines.

## Results

### Study design

**Fig. 1:**
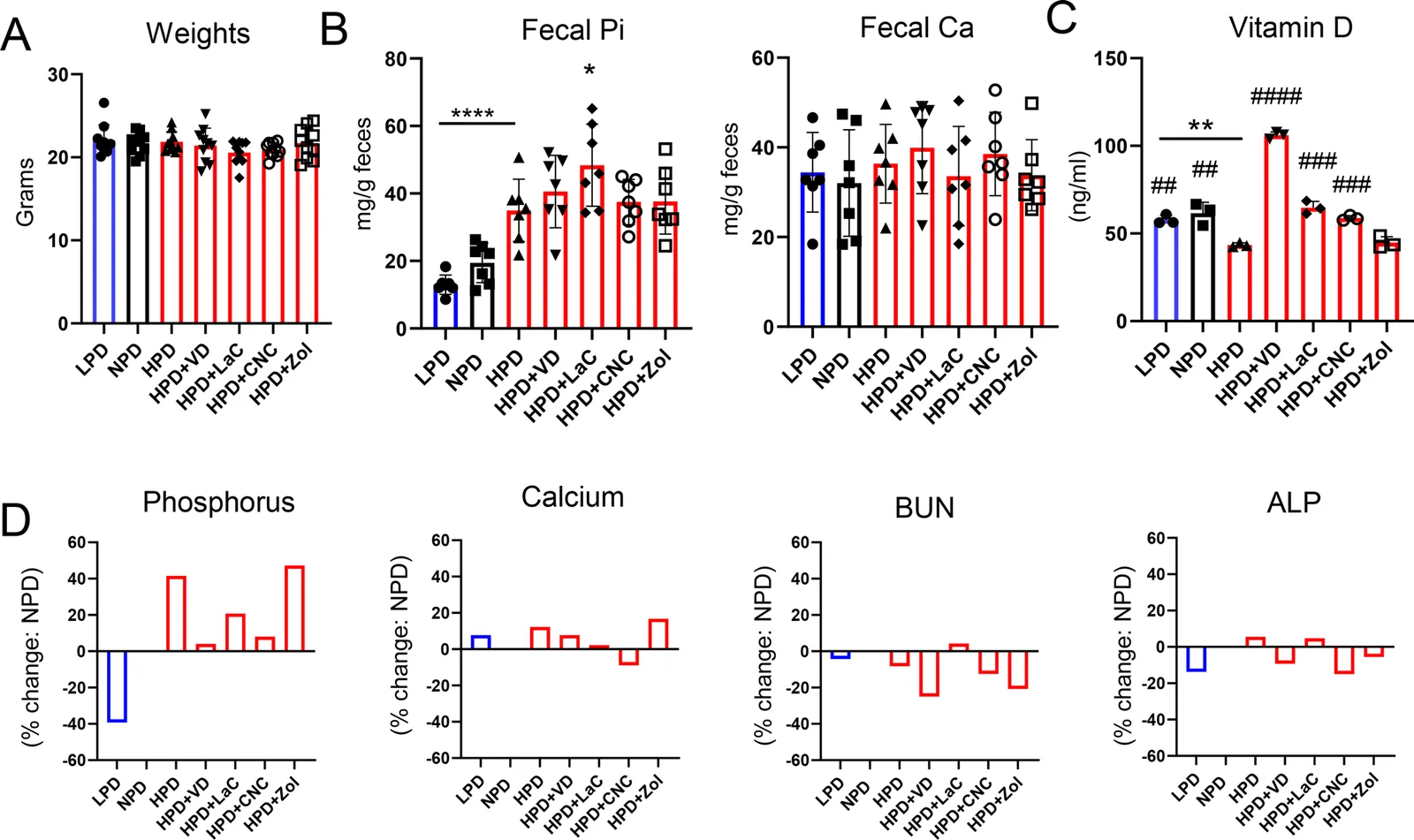
The effects of Pi diets and Pi-therapeutics on general health and systemic Pi homeostasis. (**A**) Female C57BL/6J mice (10wks) were randomized to receive LPD, NPD, HPD, HPD+VD (cholecalciferol 10IU/g incorporated in the diet), HPD+LaC (3% incorporated in the diet), HPD+CNC (100 mg/kg incorporated in the diet), or HPD+ Zol (single ip injection at the start of the study) for 5 wks and weights recorded at sacrifice. *N* = 10. (**B**) Fecal Pi and Ca were measured by ICP-MS, *N* = 7/cage. (**C**) Vitamin D (D2+D3) was measured from pooled samples (10 µl pooled serum from each diet group (*N* = 10) in technical triplicate) by ELISA. (**D**) Serum levels of phosphorus, calcium, Blood Urea nitrogen (BUN), and Alkaline Phosphatase (ALP) were measured from pooled samples as (**C**) and calculated as percent change from NPD. Data represent mean ± SD. *N* = 10/group. The overall effect of diet (LPD, NPD, HPD) was analyzed by one-way ANOVA (with test for linear trend significance). *p < 0.05, **p < 0.01, ***p < 0.001, ****p < 0.0001. Differences between treatment groups were compared to HPD by unpaired student’s t test (two-tail). #*p* < 0.05, ##*p* < 0.01, ###*p* < 0.001, ####*p* < 0.0001. Abbreviations: LPD, low-phosphate diet; NPD, normal-phosphate diet; HPD, high-phosphate diet; VD, vitamin D, LaC, Lanthanum Carbonate; CNC, Cinacalcet; Zol, zoledronic acid

Female C57BL/6J mice at 10wks of age were randomly assigned to receive LPD (0.2%), NPD (0.6%), or HPD (1.8%) diets for 5wks. These diets therefore create a 1:3, 1:1, and 3:1 Pi to Ca contents comparable to the highest and lowest percentiles described in the NHANES data [47, 48]. Additionally, four groups of mice were fed HPD plus clinically used compounds to attempt to prevent the negative effects of HPD on the skeleton or control for Pi and Ca release from the skeleton. These groups included HPD plus: 1) Vitamin D (VD; 10IU/g incorporated in the feed), 2) Lanthanum Carbonate (LaC at 3% in the feed), 3) Cinacalcet (CNC at100mg/kg in the feed), or 4) Zoledronic acid (Zol) (100 µg/kg intraperitoneal (ip) once at study start). Access to food and water was *ad libitum*. Individual mice were weighed at the completion of the time points, and no effect of diet or therapeutics was found (Fig. 1A) suggesting no gross negative health consequences.

### Pi therapeutic strategies effects on Pi excretion and serum chemistry

To determine the effects of the Pi diets and therapeutic strategies on mineral balance, Pi and Ca were measured in feces which revealed a diet dependent increase in fecal Pi content, in parallel with increased dietary intake, but no change in fecal Ca (Fig. 1B). Results confirmed the ability of LaC incorporated into the HPD to increase dietary Pi elimination, while other therapeutics resembled the increased levels of the HPD group. Ca excretion showed little difference between groups (Fig. 1B). Vitamin D (D2 and D3) was measured in serum samples that were pooled from the mice described in Fig. 1. The results identified a decrease in VD in response to sustained HPD (Fig. 1C). The mice fed HPD diet supplemented with VD had increased VD serum levels well beyond even NPD and LaC, while CNC blunted the HPD-induced decrease back to NPD levels, and Zol had no effect. Pi, Ca, and Blood Nitrogen Urea (BUN) were also measured in pooled serum samples. The average serum Pi level was dose-dependently increased in HPD mice relative to NPD or LPD, however there was little change in Ca, Blood Urea Nitrogen (BUN), or Alkaline Phosphatase (ALP) (Fig. 1D). BUN was in the range of 18 mg/dL to 25 mg/dL, considered normal for mice and suggesting clinically normal renal function. Serum Pi from HPD fed mice with VD, LaC, and CNC were more akin to NPD while mice fed and treated with HPD+Zol had serum Pi levels analogous to HPD (Fig. 1D). The average Ca level was slightly reduced by CNC and average BUN levels were lower in VD and Zol treated mice (Fig. 1D). Together, the results suggest that our therapeutic strategies are generally effective at reducing serum levels of Pi in response to sustained HPD consumption without altering Ca.

### Therapeutic strategies partially correct high dietary Pi-responsive circulating factors

**Fig. 2:**
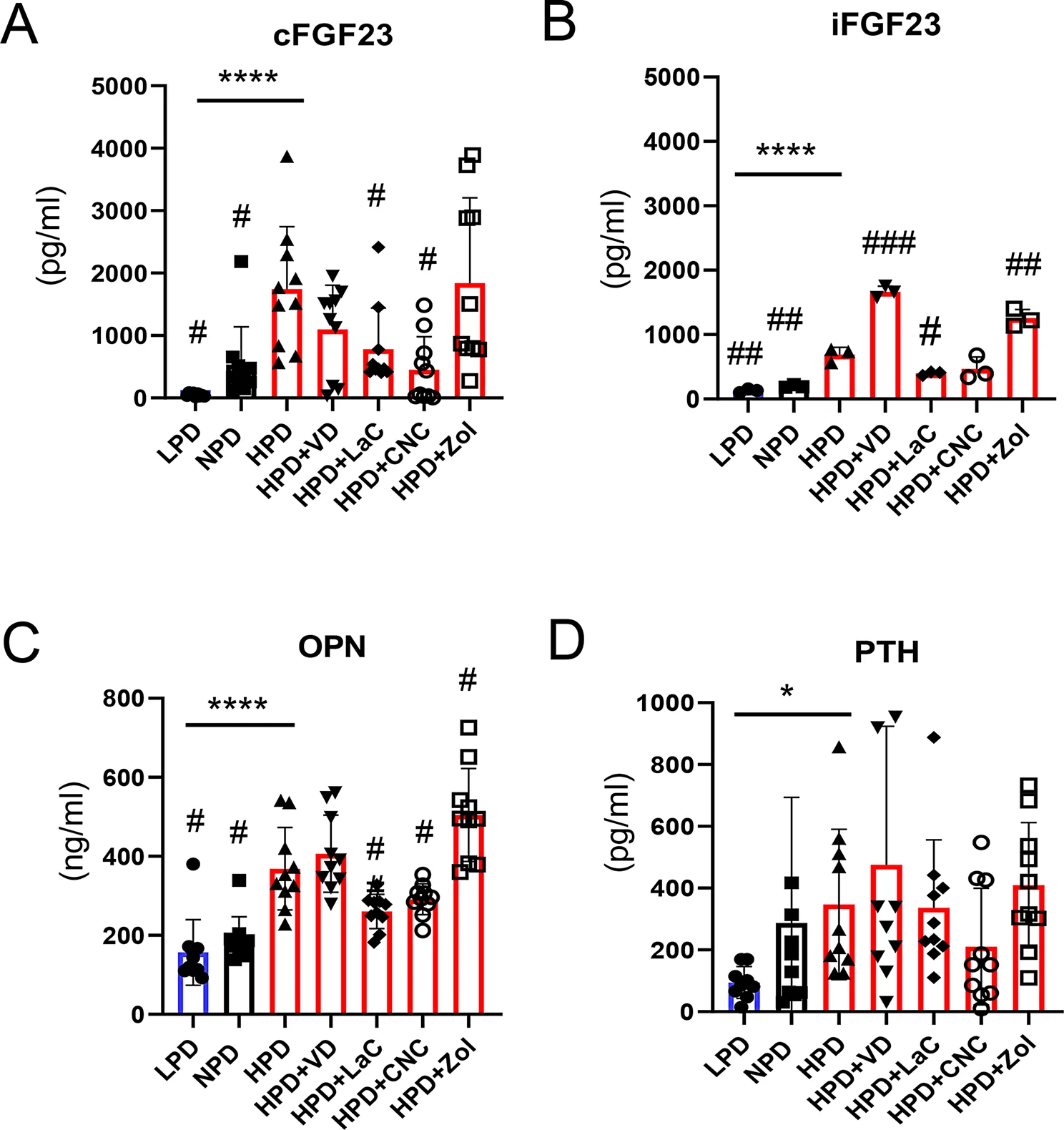
The serum from female C57BL/6J mice described in Fig. 1 was assessed by ELISA for Pi responsive endocrine factors: (**A**) c-terminal FGF23, (**B**) intact FGF23, (**C**) OPN, and (**D**) PTH. The intact FGF23 was performed on pooled samples from all mice and run in technical triplicate. The overall effect of diet (LPD, NPD, HPD) was analyzed by one-way ANOVA (with test for linear trend significance). *p < 0.05, **p < 0.01, ***p < 0.001, ****p < 0.0001. Differences between treatment groups were compared to HPD by unpaired student’s t test (two-tail). #*p* < 0.05, ##*p* < 0.01, ###*p* < 0.001, ####*p* < 0.0001. Data represent mean ± SD. *N* = 5/group

To determine the effect of the Pi-therapeutic strategies to reduce the HPD-induced changes in the levels of circulating Pi-responsive factors, serum from the mice described in Fig. 1 was analyzed by ELISA. All three serum Pi-responsive endocrine factors were significantly responsive to the base diets and strongly stimulated in mice fed HPD relative to NPD (Fig. 2). The HPD-induced increase of c-terminal FGF23 (cFGF23) was significantly blunted by Pi-lowering strategies LaC and CNC as well as reduced dietary Pi intake (LPD) (Fig. 2A). Pooled serum was also analyzed for intact FGF23 (iFGF23), and results identify the same pattern of increased circulating levels in response to HPD although slightly reduced amounts (Fig. 2B). One substantial difference between c-terminal (recognizing cleaved and intact) and intact FGF23 (only intact) was identified, whereas for HPD fed mice intact represented about 50% of circulating FGF23 in mice fed VD essential all circulating levels were intact (Fig. 2A,B). Comparable to FGF23, the HPD-induced increase in OPN was reduced by LaC and CNC, however unlike FGF23, Zol stimulated OPN in the presence of HPD (Fig. 2C). The average serum levels of PTH in the HPD+CNC fed mice were reduced by ~50% relative to HPD but due in part to the relatively wide range in the values, the difference was not statistically significant (Fig. 2D).

### Pi therapeutic strategies alter gene expression in the kidney

**Fig. 3:**
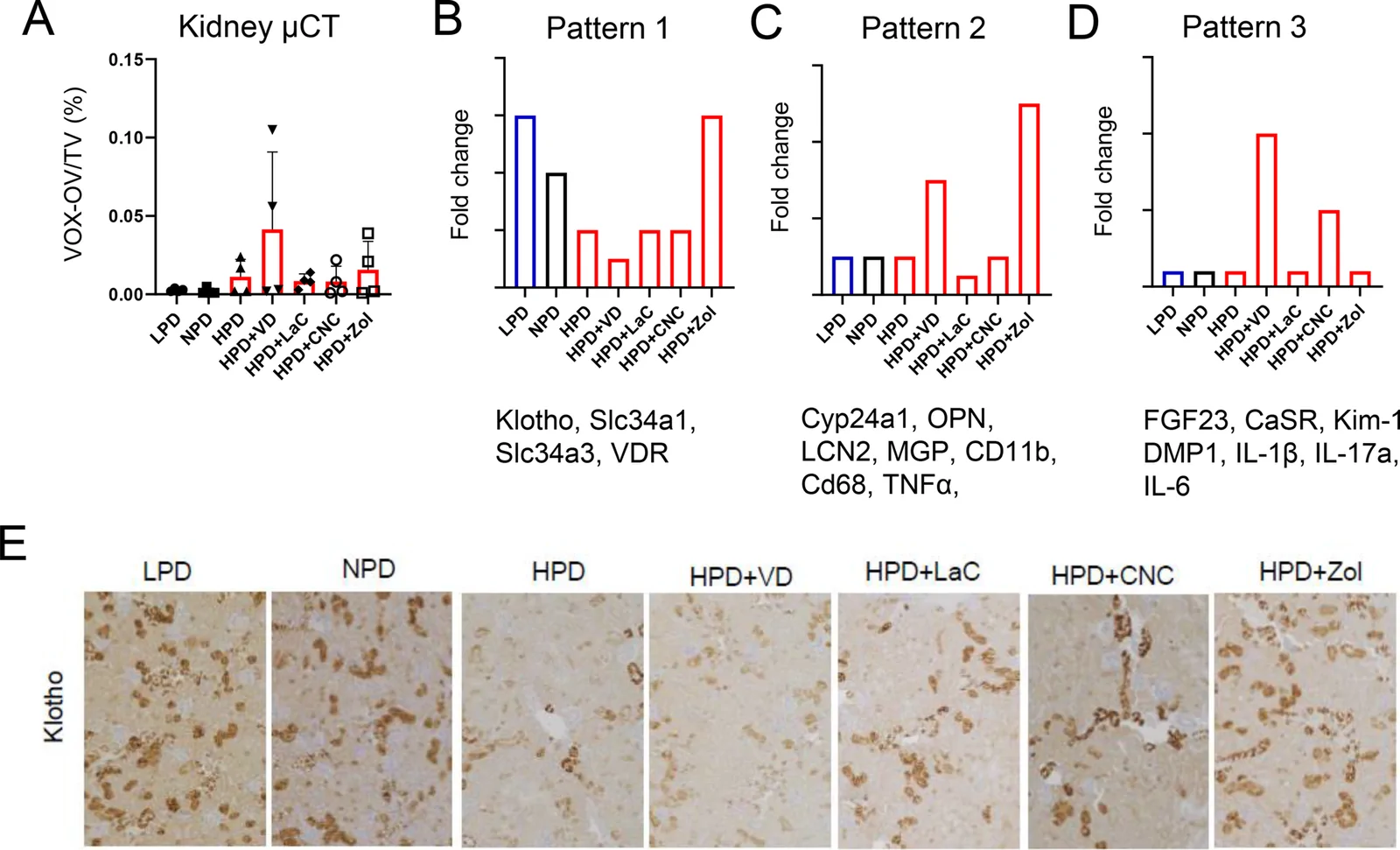
Impact of sustained changes in dietary Pi and mineral homeostasis strategies on kidney gene expression. The kidneys from female C57BL/6J, described in Fig. 1, were assessed. (**A**) The left kidney was assessed by ex vivo µCT for calcification. Data represent the mean ± SD. The overall effect of diet (LPD, NPD, HPD) was analyzed by one-way ANOVA (with test for linear trend significance) and no significant difference was found. (**B**, **C**, **D**) RNA was extracted from the right kidney and cDNA from each mouse (*N* = 10/group) were pooled. Samples were quantitated by qRT-PCR in technical triplicate. Fold change relative to NPD was calculated using ΔΔCT method. Three general patterns were identified. Expression levels of individual genes are available in Supplemental Fig. 1. (**E**) Histological sections of kidney stained by immunohistochemistry for αKlotho

To better understand the mechanism by which these therapeutic strategies alter Pi homeostatic factors and to also determine potential side effects on tissue function, calcification and gene expression were assessed from the kidneys of mice described in Fig. 1. The presence of mineral was determined by micro-computed tomography (µCT) scanning. Results suggest very low levels of mineral present (Fig. 3A). RNA was isolated from the kidney and gene expression quantitated in pooled cDNA by qRT-PCR. Different gene sets were analyzed including Pi and Ca transport, mineral regulatory factors, and related to kidney damage and inflammation. Genes with a greater than two-fold change were considered of interest. Three different patterns of gene expression were defined (Fig. 3). The expression of genes such as Klotho, VDR, and Pi co-transporters Slc34a1 and Slc34a3 were inversely responsive to dietary Pi, while only Zol blunted this HPD-induced decrease in “pattern 1” expression (Fig. 3A, Supplemental Fig.1A). The second pattern featured an increase in expression for VD and Zol and consisted of genes such as OPN (*Spp1*) and Cd11b both expressed by multiple cell types including macrophages (Fig. 3C; Supplemental Fig. 1B). Of note, VD increased Cyp24a1 expression as would be predicted, confirming the activity of VD in the HPD. A third group of genes “pattern 3” showed a minimal effect from the base diets but a strong increase to the HPD+VD and HPD+CNC conditions and included both mineral regulating genes, such as FGF23 and MGP, and inflammatory markers Il-1b, IL-17a, and IL-6 among others (Fig. 3B; Supplemental Fig. 1C). The diet responsive gene expression pattern of Klotho was confirmed by immunohistochemistry (Fig. 3E).

### The HPD-driven decrease in trabecular bone volume is not corrected by Pi therapeutic strategies

**Fig. 4:**
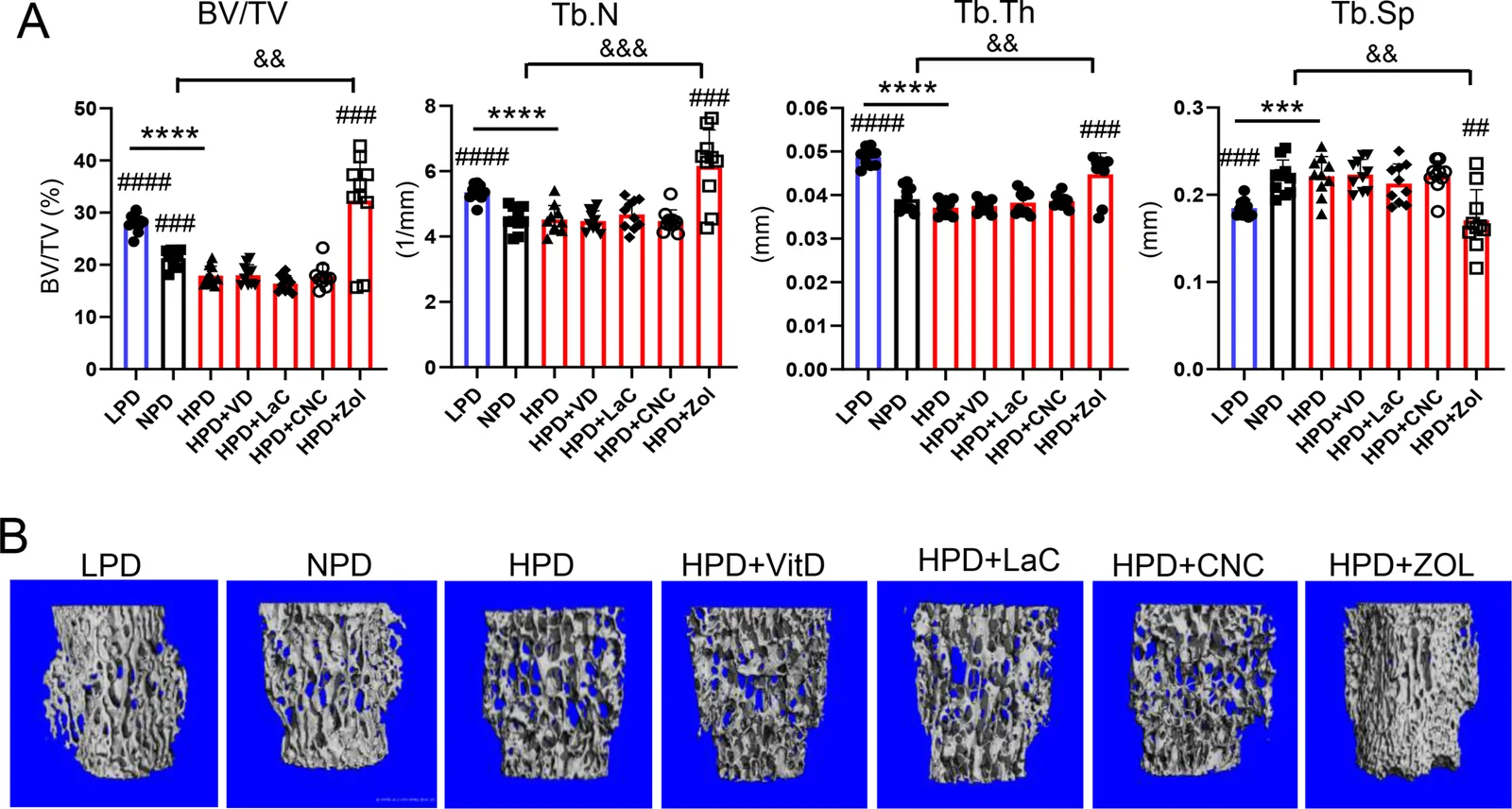
The vertebrae from female C57BL/6J mice described in Fig. 1 were assessed by µCT quantification of bone volume fraction for: (**A**) spine BV/TV (bone volume/tissue volume), Tb.N (trabecular number), Tb.Th (trabecular Thickness), and Tb.Sp (trabecular spacing). (**B**) Representative µCT images of spine trabecular. The overall effect of diet (LPD, NPD, HPD) was analyzed by one-way ANOVA (with test for linear trend significance). *p < 0.05, **p < 0.01, ***p < 0.001, ****p < 0.0001. Differences between treatment groups were compared to HPD by unpaired student’s t test (two-tail). #*p* < 0.05, ##*p* < 0.01, ###*p* < 0.001, ####*p* < 0.0001. Data represent mean ± SD. *N* = 5/group

To determine the ability of our Pi homeostasis management strategies to blunt the negative impacts of a HPD on skeletal metabolism, the spines from the female mice described in Figure 1 were assessed by ex vivo µCT for changes in trabecular bone volume. The amount of Pi in the base diets negatively correlated with vertebral trabecular volume (Fig. 4A). Specific indices of trabecular bone including, trabecular number (Tb.N) and trabecular thickness (Tb.Th), agreed with BV/TV showing a Pi diet-dependent negative correlation while trabecular spacing (Tb.Sp) was positively correlated (Fig. 4A). Manipulating the Pi homeostasis network with either VD, LaC, or CNC did not alter the HPD induced reduction in trabecular bone volume or associated indices (Fig. 4A). However, Zol significantly blocked the HPD-induced decrease in all bone indices and generated a significant additive benefit above NPD (Fig. 4A). Representative µCT images of vertebral bone reflect the quantification results (Fig. 4B). LPD was effective at increasing trabecular bone volume similar to Zol after 5wks. Together, the results suggest that even under conditions in which serum Pi and/or Pi homeostatic endocrine factors were generally normalized, mice fed HPD still suffered a decrease in trabecular bone volume.

### Divergent effects of Pi-therapeutics on cortical bone volume and porosity

**Fig. 5:**
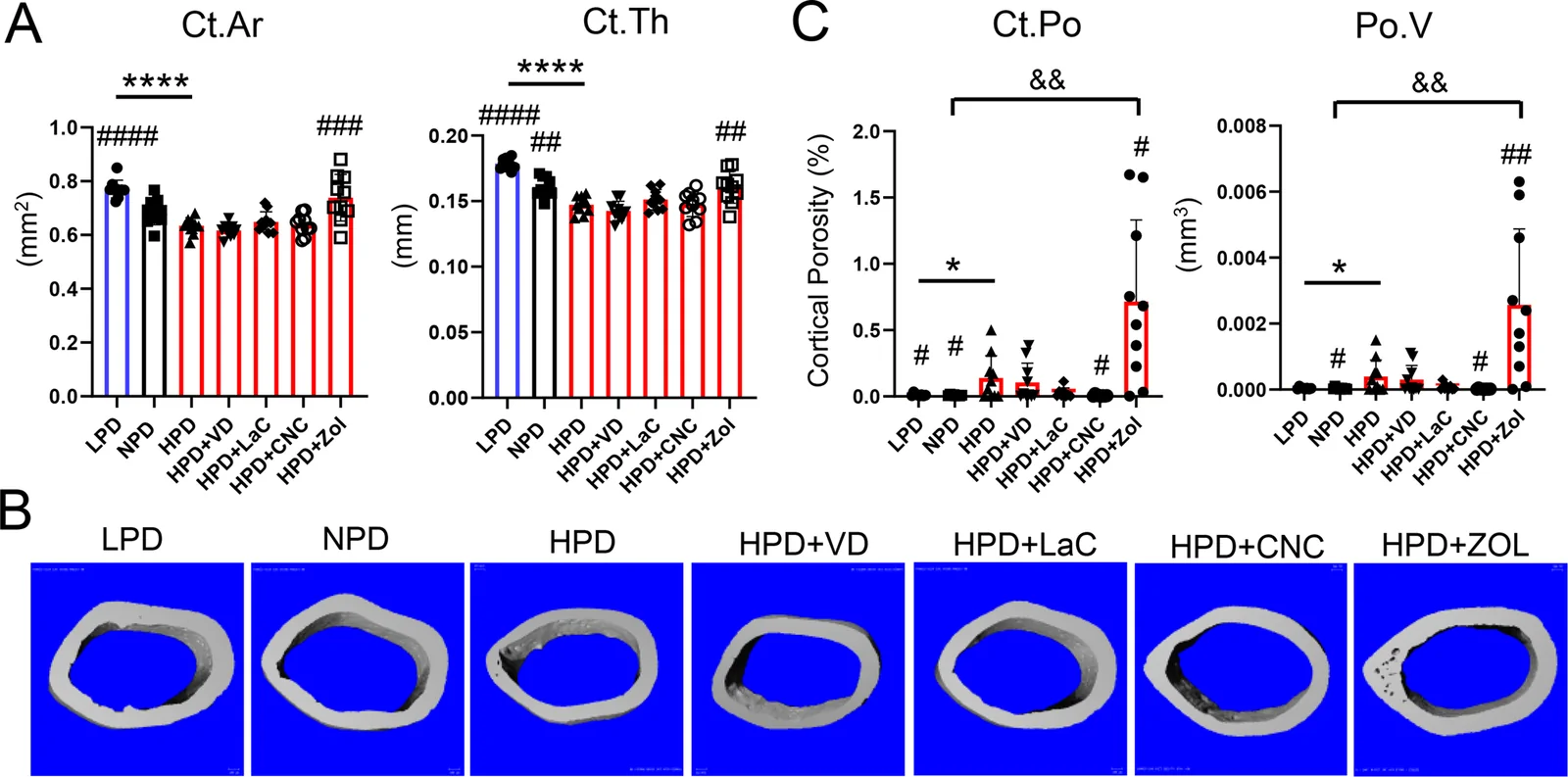
The femurs from female C57BL/6J mice described in Fig. 1 were assessed by µCT quantification of bone volume fraction for: (**A**) Cortical area (Ct.Ar) and cortical thickness (Ct.Th) and (**B**) Cortical porosity (Ct.Po) and pore volume (Po.V). (**C**) Representative µCT images of femur cortical bone. The overall effect of diet (LPD, NPD, HPD) was analyzed by one-way ANOVA (with test for linear trend significance). *p < 0.05, **p < 0.01, ***p < 0.001, ****p < 0.0001. Differences between treatment groups were compared to HPD by unpaired student’s t test (two-tail). #*p* < 0.05, ##*p* < 0.01, ###*p* < 0.001, ####*p* < 0.0001. Data represent mean ± SD. *N* = 5/group

The response of cortical bone volume and porosity to the Pi diets and therapeutic strategies was therefore measured in femur. There was a Pi-diet dependent effect on femoral cortical bone, with HPD generating reduced bone volume and LPD increasing bone volume relative to NPD (Fig. 5A) and reflected in the µCT images (Fig. 5B). The Pi therapeutic strategies did not substantially alter the HPD-driven reduction in cortical volume. Zol blunted the negative impact of HPD on cortical bone volume however, unlike the substantial Zol driven increase in trabecular bone above NPD values, there was no added benefit of Zol to cortical bone. Cortical porosity was also assessed and was positively correlated with increasing dietary Pi consumption (Fig. 5C). However, unlike other trabecular and cortical indices, the HPD-induced increase in cortical porosity was significantly reduced by CNC. Interestingly, the HPD induced increase in cortical porosity was exacerbated by Zol. Further, the increase in porosity was due to an increase in pore volume (Fig. 5C) as opposed to pore number (Supplemental Fig. 1E) and reflected by µCT images (Supplemental Fig. 1F). Combined with data from serum analyses, the results suggest that correcting Pi homeostasis and associated endocrine factors in response to sustained high Pi consumption does not reduce HPD-induced cortical bone loss but may blunt an increase in porosity.

### Serum markers of bone metabolism are partially corrected by Pi therapeutics

**Fig. 6:**
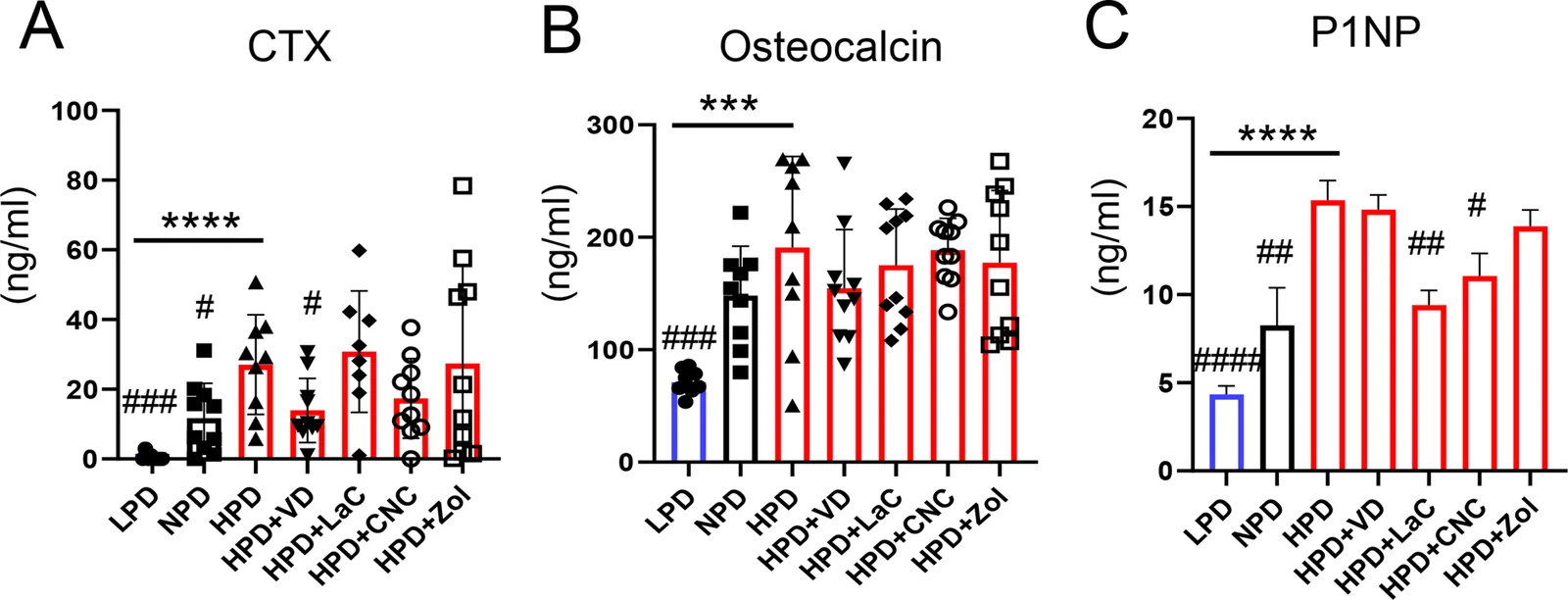
Serum was assessed by ELISA for bone metabolism markers (**A**) Collagen type-1 C-Telopeptide (CTX), (**B**) osteocalcin, and (**C**) Procollagen type-1 intact N-terminal propeptide (P1NP). The overall effect of diet (LPD, NPD, HPD) was analyzed by one-way ANOVA (with test for linear trend significance). *p < 0.05, **p < 0.01, ***p < 0.001, ****p < 0.0001. Differences between treatment groups were compared to HPD by unpaired student’s t test (two-tail). #*p* < 0.05, ##*p* < 0.01, ###*p* < 0.001, ####*p* < 0.0001. Data represent mean ± SD. *N* = 5/group

Measurement of serum bone metabolism markers by ELISA identified a Pi-diet induced increase in bone resorption marker cross-linked C-telopeptide of type I collagen (CTX) (Fig. 6A). Only VD significantly reduced levels compared to HPD. Likewise, the formation marker osteocalcin was dose dependently increased in response to dietary Pi although none of the Pi therapeutics or Zol reduced serum levels below HPD levels (Fig. 6B). Another marker of bone formation, P1NP, was measured in pooled serum and was found to be dose dependent and positively correlated with dietary Pi intake (Fig. 6C). In contrast to osteocalcin, both LaC and CNC reduced the HPD induced increase in P1NP. The results identify an increase in bone metabolism with an increasing dietary Pi to Ca ratio and that although certain markers of resorption and formation can be altered by Pi therapeutics, they do not necessarily reflect the changes in bone volume after 5wks. The difference could be a result of this single time point and that some changes in bone may have occurred earlier in the response.

### A nutritional strategy to mitigate HPD-induced health consequences in female mice

In addition to therapeutic strategies, a dietary strategy was also used to attempt to reduce or blunt the negative consequences of a HPD on bone. Two Ca balanced diets were used to match Pi in the HPD to create a high Ca-Pi diet (HCPD) or decreased the Ca content to match Pi in the LPD to create a low Ca-Pi diet (LCPD) as previously described [28]. Female C57BL/6J mice, 10wks of age, were randomized to one of the five Ca-Pi diets for 10wks. Data associated with LPD, NPD, and HPD has been as previously reported [20]. The female mice had little change in weights on any of the diets (Table 1). Serum Pi was not statistically different, possibly due to a wider than normal range, although the average increased with increasing dietary Pi. Serum Ca levels were not different between groups. Vitamin D was measured in pooled samples and did not show dramatic differences, although the low Ca diet (LCPD) generated the highest levels (Table 1). FGF23 and OPN were increased by increasing dietary Pi, and the addition of high Ca to the high Pi diet normalized the serum levels (Table 1). Collectively, the results suggest that when serum factors were responsive to the amount of Pi in the diet, they were generally corrected by balancing the amount of Ca intake with Pi.

**Table 1:** Serum measurements from female mice (+/- Ca)

Female	LCPD	LPD	NPD	HPD	HCPD
Weight (g)	21.6 ± 1.6	20.9 ± 1.4	21.3 ± 2.0	20.0 ± 2.1	19.3 ± 4.1
Pi (mg/dl)	12.7 ± 6.4	12.1 ± 3.5	19.3 ± 6.9	22.3 ± 9.9	19.0 ± 12.0
25(OH)D (ng/ml)	101.1	75.3	88.4	76.2	88.5
FGF23 (pg/ml)	381 ± 87##	197 ± 68	507 ± 143	1854 ± 883	742 ± 347#
OPN (ng/ml)	212 ± 40	303 ± 133	242 ± 26	444 ± 37	315 ± 91#
CTX (ng/ml)	34.1 ± 27.0	31.8 ± 22.1	24.1 ± 11.5	45.2 ± 32.5	32.1 ± 17.4
Osteocalcin (ng/ml)	41.3 ± 20.6	28.4 ± 20.6	56.9 ± 25.9	107.1 ± 62.8	68.6 ± 39.1

Serum from female mice at 10wks of age fed diets for 10wks was analyzed by ELISA. LCPD was compared to LPD and HCPD to HPD by student’s t test. #*p* < 0.05, ##*p* < 0.01, ###*p* < 0.001, ####*p* < 0.0001. Vitamin D was measured from pooled samples. Data represent mean ± SD. *N* = 8–10/group. Data from LPD, NPD, HPD mice has been previously reported (Roberts et al., 2023)

### Balancing dietary calcium does not correct the HPD-induced reduction in bone volume in female mice but improves cortical porosity

**Fig. 7:**
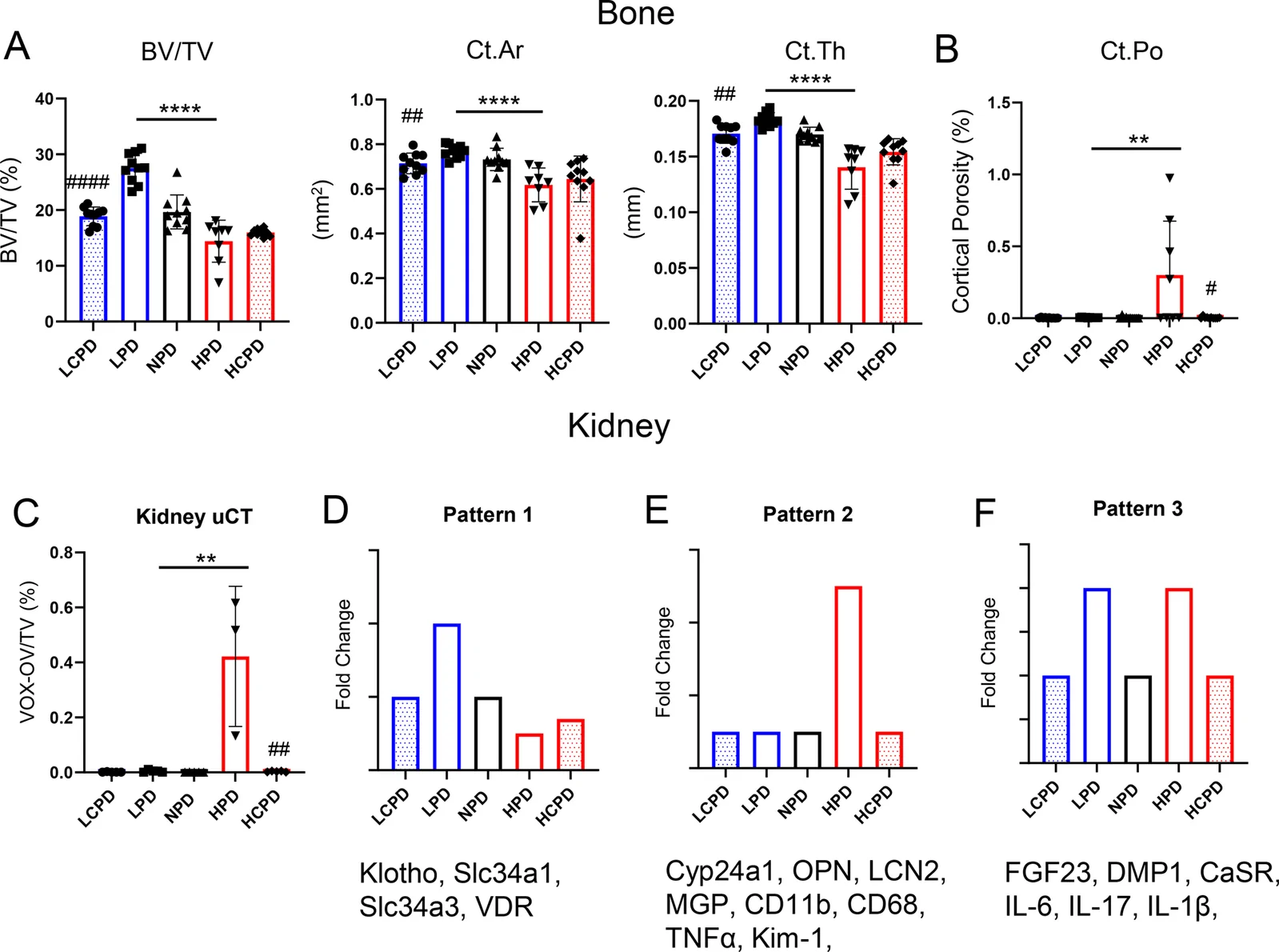
Female C57BL/6J mice at 10wks of age were fed diets as indicated for 10wks and spine and femur harvested. Ex vivo µCT was used to measure changes in (**A**) BT/TV, CT.Ar and Ct.Th and (**B**) Cortical porosity (Ct.Po). (**C**) The left kidney was assessed by µCT. (**D**, **E**, **F**) RNA was extracted from the right kidney and quantitated by qRT-PCR and cDNA from each mouse (*N* = 8–10/group) were pooled and pooled samples run in technical triplicate, and three general patterns were identified. Expression levels of individual genes are available in Supplemental Fig. 2. The overall effect of diet (LPD, NPD, HPD) was analyzed by one-way ANOVA (with test for linear trend significance). *p < 0.05, **p < 0.01, ***p < 0.001, ****p < 0.0001. Differences between LPD and LCPD and between HPD and HCPD were compared by unpaired student’s t test (two-tail). #*p* < 0.05, ##*p* < 0.01, ###*p* < 0.001, ####*p* < 0.0001. Data represent mean ± SD. *N* = 8–10/group

Changes in vertebral BV/TV and cortical area and thickness were assessed by µCT. In female mice, there was a dose-dependent inverse relationship between dietary Pi intake and vertebral trabecular and cortical bone volume as previously described for this cohort [20]. The addition of Ca to the diet (HCPD) did not correct the HPD-induced decrease in bone volume in opposition to serum parameters. Additionally, the positive effect on bone volume of LPD was lost by decreasing Ca (LCPD) (Fig. 7A), also in general opposition to the serum indices. Cortical porosity was also measured by µCT and an increase to HPD was detected after 10wks of diet (Fig. 7B), similar to the 5 wk timepoint reported above and similarly due to a change in pore volume and not number (Supplemental Fig. 2A,B). The HPD-driven increase was corrected by the supplementing HPD with Ca (Fig. 7B). Bone metabolism markers CTX and Osteocalcin were both elevated in response to HPD as previously reported (Table 1 and [20]) and not significantly lowered by HCPD. The results suggest that although the addition of elevated Ca to a high Pi diet can correct most of the systemic responses, the benefit of high Ca to bone is limited to a reduction of the HPD-induced increase in cortical porosity.

### Balancing dietary calcium in females corrects HPD-induced changes in gene expression in the kidney

Assessment of the kidney from mice described in Table 1 by µCT identified an increase in calcification in the HPD mice (Fig. 7C), in contrast to the female mice fed HPD for 5wks (Fig. 3). Changes in kidney gene expression were also assessed. Three general patterns of gene expression were detected (Fig. 7 and individual genes in Supplemental Fig. 2C,D,E). Pattern 1 resembled Pattern 1 of Fig. 4 with the Pi diets generating a diet-depending decrease in expression and included similar genes such as Klotho, the Pi transporters, and VDR (Fig. 7D). This decrease was only partially rescued by the addition of high Ca to the HPD, while the lowering of Ca, (LCPD) generally reduced any LPD driven increase in expression. Pattern 2 characterized a group of genes that were stimulated in response to HPD and were corrected with the addition of Ca (HCPD) (Fig. 7E). The function of this group encompassed VD regulation and inflammation/injury. Interestingly, Cyp24a1 was the only gene of this group in which the HPD-induced increase in gene expression was not reduced with the balancing of Ca (Supplemental Fig. 2C). Pattern 3 was characterized by genes that had increased expression in both the LPD and HPD groups and consisted mainly of inflammatory genes and mineral regulating genes/proteins (Fig. 7F). Taken together, the results suggest that most Pi responsive kidney expressed genes are more sensitive to Pi:Ca ratio than the absolute amount of Pi in the diet.

### A nutritional strategy to mitigate HPD-induced health consequences in male mice

Previous studies have suggested a sex difference in renal calcium handling [58–61] and therefore the effects of balancing dietary Pi and Ca in both female and male mice were investigated. Male C57BL/6J mice at 10wks of age were randomized to the Pi/Ca diets for 5wks or institutional “house” chow. Male mice fed LPD had increased weight and HPD fed mice had deceased weight compared to NPD as reported previously [28] and (Table 2). Balancing dietary Ca intake to Pi corrected the HPD-induced changes in weight. Male mice exhibited an increase in serum Pi with increasing dietary intake which was returned to NPD levels with the addition of high dietary Ca [28] (Table 2). Serum Ca levels were not different between groups. Vitamin D was measured in pooled samples and did not show substantial differences, although as with female mice, the low Ca diet (LCPD) generated the highest circulating levels (Table 2). FGF23 and OPN were increased by increasing dietary Pi and the addition of high Ca to the high Pi diet normalized the serum levels (Table 2), and as previously reported [28].

**Table 2:** Serum measurements from male mice (+/- Ca)

Male	House	LCPD	LPD	NPD	HPD	HCPD
Weight (g)	27.3 ± 2.9	26.2 ± 1.2####	34.4 ± 1.5	27.6 ± 0.9	23.2 ± 1.4	28.4 ± 2.7##
Pi (mg/dl)	9.4 ± 2.9	11.4 ± 2.1	8.5 ± 0.8	12.3 ± 1.4	12.5 ± 1.4	10.1 ± 1.3#
Ca (mg/dl)	9.5 ± 1.2	8.3 ± 0.6	8.4 ± 0.0	8.9 ± 0.8	8.6 ± 1.0	8.2 ± 0.22
25(OH)D (ng/ml)		106.9	62.3	86.6	73.6	83.6
FGF23 (pg/ml)	206 ± 34	209 ± 70	198 ± 90	277 ± 15	1303 ± 308	358 ± 100###
OPN (ng/ml)	212 ± 40	386 ± 69###	188 ± 39	235 ± 42	743 ± 142	260 ± 49####
PTH (pg/ml)	114 ± 58	131 ± 74	133 ± 103	260 ± 181	355 ± 244	396 ± 147
CTX (ng/ml)		59.8 ± 32.0	26.6 ± 5.0	26.1 ± 15.0	52.6 ± 17.4	31.0 ± 13.1
Osteocalcin (ng/ml)		33.6 ± 16.3	24.9 ± 3.3	26.6 ± 6.1	21.3 ± 5.5	37.9 ± 9.5##
P1NP (ng/ml)		26.0 ± 7.0	25.9 ± 11.5	18.9 ± 1.0	17.0 ± 4.5	16.5 ± 3.4

Serum from male mice at 10wks of age fed diets for 5wks was analyzed by ELISA. LCPD was compared to LPD and HCPD to HPD by student’s t test. #*p* < 0.05, ##*p* < 0.01, ###*p* < 0.001, ####*p* < 0.0001. Vitamin D was measured from pooled samples. Data represent mean ± SD. *N* = 5/group. Data from Pi diets has been previously reported (Gutierrez et al., 2020)

### Balancing dietary calcium only partially corrects the HPD-induced reduction in bone volume in male mice

**Fig. 8:**
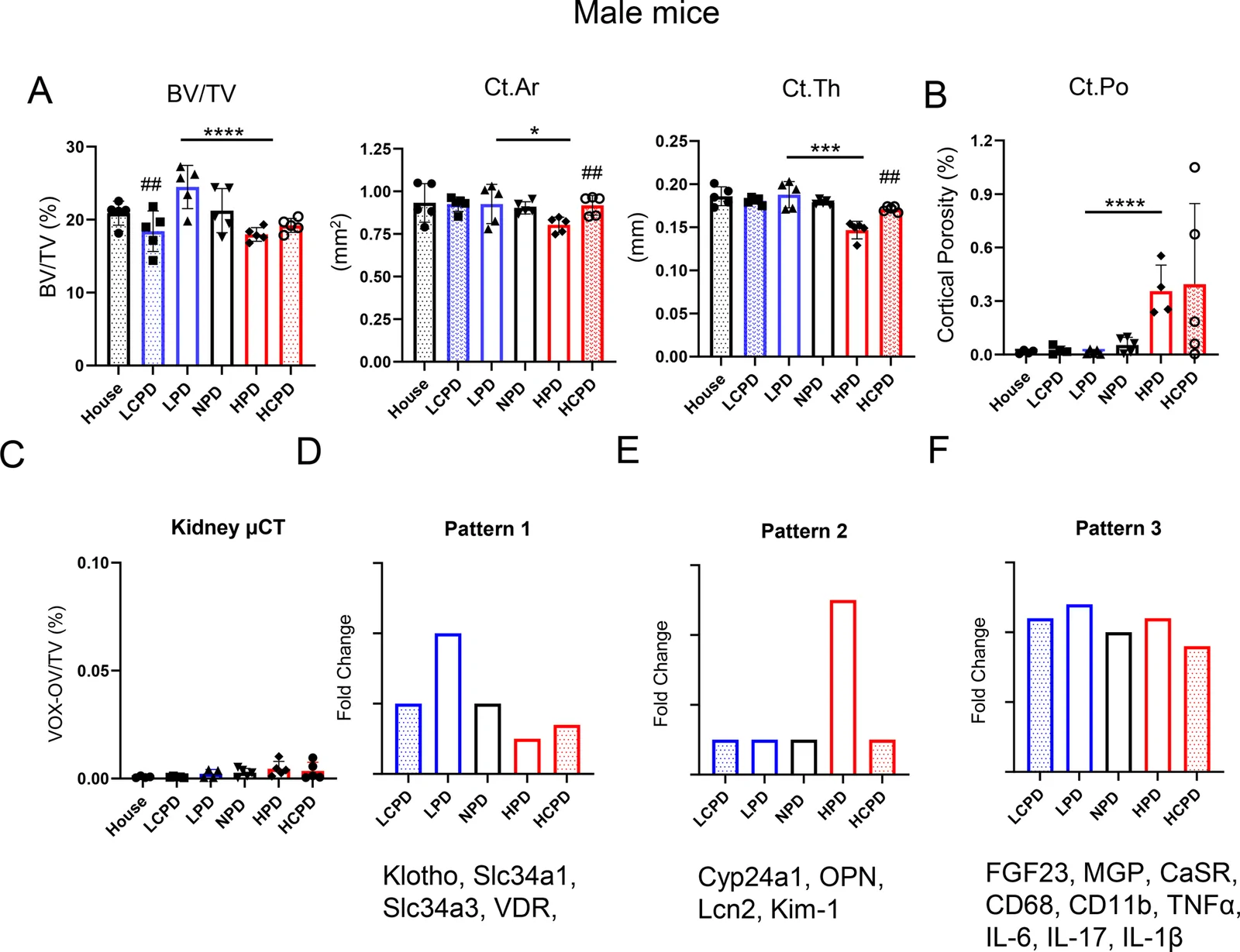
Male C57BL/6J mice at 10wks of age were fed diets as indicated for 5wks and spine and femur harvested. Ex vivo µCT was used to measure changes in (**A**) BT/TV, CT.Ar and Ct.Th and (**B**) Cortical porosity (Ct.Po). (**C**) The left kidney was assessed by µCT. (D,E,F) RNA was extracted from the right kidney and quantitated by qRT-PCR and cDNA from each mouse (*N* = 5/group) were pooled and pooled samples run in technical triplicate, and three general patterns were identified expression levels of individual genes are available in Supplemental Fig. 3. The overall effect of diet (LPD, NPD, HPD) was analyzed by one-way ANOVA (with test for linear trend significance). *p < 0.05, **p < 0.01, ***p < 0.001, ****p < 0.0001. Differences between LPD and LCPD and between HPD and HCPD were compared by unpaired student’s t test (two-tail). #*p* < 0.05, ##*p* < 0.01, ###*p* < 0.001, ####*p* < 0.0001. Data represent mean ± SD. *N* = 5/group

Male mice fed HPD had decreased vertebral trabecular BV/TV and this was not corrected with the Ca added diet (HCPD). Decreasing dietary Ca eliminated the trabecular bone gain associated with LPD (Fig. 8A) in agreement with data from female mice. Interestingly, in male mice, the high Ca balanced diet significantly blunted the HPD-induced decrease in cortical bone volume however the lower Ca diet did not reduce cortical indices below LPD. In contrast to the results in female mice, addition of high Ca to the HPD did not correct the HPD-induced increase in cortical porosity (Fig. 8B) which was again due to an increase in pore volume (Supplemental Fig. 3A,B). Mice fed the grain based “house” diet were similar to NPD for all indices (Fig. 8A,B). While bone resorption marker CTX was elevated, formation markers osteocalcin and P1NP were generally unremarkable (Table 2), as previously reported [28]. Collectively, the results suggest that in male mice, HPD-induced trabecular bone loss is responsive to the absolute amount of Pi in the diet and the trabecular gain associated with a low Pi diet requires sufficient Ca. Further, cortical bone volume gain or loss is more responsive to the ratio of Pi to Ca, whereas in female mice the absolute amount of dietary Pi was the predominant determinant of both trabecular and cortical bone volume.

### Balancing dietary calcium in males corrects HPD-induced changes in gene expression in the kidney

Assessment of the kidney from mice described in Table 2 by µCT identified no increase in calcification in the HPD mice (Fig. 8C), in agreement with the female mice fed HPD for 5wks (Fig. 3) but in contrast to the 10 wk diet mice (Fig. 7). Changes in kidney gene expression were also assessed. Three general patterns of gene expression were detected (Fig. 8) and individual genes in (Supplemental Fig. 3C,D,E). Pattern 1 featured a diet dependent decrease in expression of Klotho, the Pi transporters Slc34a1 and Slc34a3, and VDR (Fig. 8D), paralleling Pattern 1 of Fig. 4. The HPD-induced decrease was only partially rescued by the addition of high Ca to the HPD, while the lowering of Ca, (LCPD) generally reduced any LPD driven increase in expression. Pattern 2 was characterized by a group of genes that were stimulated in response to HPD, and this increase was corrected by the addition of dietary Ca (HCPD) (Fig. 8E). The function of this group encompassed injury and inflammation. Mirroring the studies above, Cyp24a1 was elevated by dietary Pi and was corrected by increasing dietary Ca (Supplemental Fig. 3C). Pattern 3 was characterized by genes that did not have a strong diet-dependent response (Fig. 8F). Taken together, the results suggest that most Pi responsive kidney expressed genes are more sensitive to Pi:Ca ratio than the absolute amount of Pi in the diet.

## Discussion

Growing evidence demonstrates that chronic consumption of a diet high in Pi-additives, even with normal kidney function, negatively affects bone mineral metabolism and contributes to kidney and cardiovascular disease. In the context of healthy kidney function, dietary intake primarily drives changes in serum Pi levels, which remain elevated up to 8 hours [11, 12, 62]. This is particularly relevant in the U.S. where national dietary surveys indicate that average adult Pi intake nearly doubles the FAD/IOM recommended daily allowance of 700 mg/d [48], with those in the ninetieth percentile, particularly males, consuming between 2500-3000 mg/d [18, 63]. Much of this excess intake is attributed to UPFs, notably “enhanced” meats and fast foods [64], that contribute over 1000 mg/d of Pi to daily consumption [65]. These intake estimates likely underrepresent actual consumption due nutrient databases not fully accounting for Pi as a food additive [14, 63, 66]. Further, Pi additives are in the form of inorganic salts, which are absorbed more efficiently (80–100%) than naturally occurring organic phosphates (40–60%), which require enzymatic digestion [14, 67]. Despite widespread dietary exposure, few studies have evaluated interventions that mitigate the potential harmful effects of chronic high Pi additive intake in the context of healthy kidney function.

Results from healthy female and male mice fed low (LPD), normal (NPD), or high (HPD) Pi-additive diets for 5 or 10wks identified a positive correlation between increasing dietary Pi-additives intake, serum levels of Pi-responsive endocrine factors, FGF23, PTH, and OPN, while levels of Vitamin D2/D3 decreased. These results are as expected from previous studies and did not differ substantially between female and male mice. The varying Pi diets did not alter weights in female mice in agreement with a previous study using these diets that was extended to 20wks of diet [20]. However, male mice demonstrated an inverse relationship between weight and Pi consumption which was normalized when Ca was matched to Pi in the diets [28]. The results are in general agreement with a number of other studies [29, 68–71] and together, suggest a sex-dependent ability to metabolically compensate for differences in Pi consumption.

The increased ratio of dietary Pi to Ca resulted in a decrease in trabecular and cortical bone volume relative to NPD which align with other rodent studies demonstrating that high Pi and moderate calcium diets negatively [14, 16, 19, 20, 23, 26, 27, 29]. In contrast, a reduced Pi diet (LPD) increased these bone indices relative to control (NPD). This study also identified an increase in cortical porosity in response to HPD. The HPD-driven increase in cortical porosity correlated with pore volume but not pore number. In addition to skeletal changes, the HPD significantly altered kidney gene expression. In agreement with other studies, a sustained HPD generated a decrease in expression of Pi homeostasis genes such as klotho [72] and Pi-transporters Slc34a1 and Slc34a3 [73] while upregulating immune response and tissue injury related genes LCN2 (Lipocalin-2, *NGAL*), OPN (*spp1, eta-1*), and Kim-1 (Kidney injury molecule-1, *HAVcr-1*) [74–79]. Although long-term human dietary studies are limited, and data related to bone volume is lacking, similar changes in dietary Pi-induced endocrine factors have been reported between mice and humans [19, 28]. Collectively, these results strongly support the conclusion that the intake of sustained excess Pi, in the context of stable Ca levels, negatively impacts bone health and impacts gene expression related to inflammation and mineralization in the kidney.

One mechanism by which high Pi-additive diets might influence bone, involves elevated serum Pi, which may directly disrupt bone metabolism. In the context of CKD, clinicians commonly use an orally administered Pi-binder such as Lanthanum Carbonate (LaC). LaC, marketed as Fosrenol, is a safe, relatively well tolerated Pi-binder for the treatment of hyperphosphatemia [80–84]. Taken orally with meals, it effectively binds dietary Pi in the intestine, reducing gastrointestinal tract absorption and increasing fecal excreting as insoluble LaC [85, 86] without inducing hypercalcemia [87, 88]. Although multiple studies have demonstrated that LaC reduces serum Pi in CKD, its ability to reduce circulating FGF23 levels are unclear. With some reporting declines [89, 90] and others reporting no effect [91–94]. A recent Meta-analysis concluded that LaC reduced all-cause mortality in hemodialysis patients but does not decrease the risk of cardiovascular events [95] and had no adverse effects on bone [96]. In this study, LaC successfully increased fecal Pi excretion and reduced circulating FGF23 and OPN levels in parallel with serum Pi. LaC did not alter HPD-induced PTH in agreement with studies in humans with compromised kidney function [82, 97]. LaC also had no effect on HPD-induced bone loss or changes in kidney gene expression. These results suggest that normalizing serum Pi levels alone does not prevent HPD-induced systemic functional consequences or changes in Pi-responsive circulating factors. Thus, serum Pi does not appear to be the primary driver of bone pathology in the context of high Pi additive consumption.

A second proposed mechanism for high Pi-additive induced bone loss is sustained high serum PTH levels. This generates a secondary hyperparathyroidism, which has been associated with bone loss through stimulation of osteoclastic bone resorption [98, 99]. High Pi-additive consumption can increase serum PTH through a transient hypocalcemia [24, 100], or reports suggest that Pi can directly stimulate PTH levels [101, 102]. Cinacalcet (CNC), an oral calcimimetic (Sensipar), reduces serum PTH, Ca, and Pi in both humans and rodent and used for the management of primary or secondary hyperparathyroidism usually in the context of kidney dysfunction [103–109]. PTH is increased by hyperphosphatemia [110], which can occur from increased dietary Pi intake or in CKD, resulting in secondary hyperparathyroidism [111]. PTH then acts to decrease serum Pi by decreasing Pi-transporters in the kidney which decreases Pi reabsorption [40]. In addition to PTH, CNC has been reported to reduce FGF23 [107, 112–115], which consequently can result in hyperphosphatemia [116, 117]. Studies using rodent CKD models have identified that CNC and the calcimimetic Etelcalcetide (Parsabiv) can improve bone volume and decrease CKD-induced cortical porosity [118–120]. In healthy mice, CNC significantly reduced HPD-induced hyperphosphatemic-like response, and serum FGF23 and OPN levels. Although the wide range of values did not generate a significant decrease in HPD-induced PTH, CNC did reduce circulating PTH levels by ~50%. CNC also blocked the HPD-induced decrease in circulating Vitamin D2/D3 levels. CNC did not substantially alter HPD-induced kidney gene expression, although it did exacerbate some of the HPD-driven response. Although CNC altered the systemic endocrine response to HPD, surprisingly it had no effect on HPD-induced loss of bone volume. CNC did however significantly reduce the HPD-induced increase in cortical porosity. These findings, taken with the LaC results, suggest that FGF23 or PTH do not play essential roles in mediating HPD changes in bone volume but correlate with changes in cortical porosity in the context of chronic Pi consumption.

A third potential explanation for HPD-induced bone loss posits that a high concentration of Pi in the gut reduces Ca absorption [23]. Short-term studies (<1 wk), demonstrate that serum Ca levels drop in response to oral phosphate [110]. Vitamin D supplementation is an established strategy to increase Ca absorption in the gut [121, 122], although outside of VD deficiency and compromised kidney function, the benefit to bone remains controversial [123]. VD supplementation is widespread, with an estimated 20–30% of adults in the US taking a VD supplement and 40% in a multi-vitamin containing VD [124]. However, the impact of VD supplementation in the context of a HPD remains relatively unknown. Supplementation of the HPD with VD for 5wks increased serum D2/D3 levels and decreased serum Pi but did not alter serum Ca or significantly affect PTH, OPN, cFGF23. It did increase intact FGF23, and in agreement with previous studies, increased Cyp24a1 expression in the kidney while decreasing Cyp27b1 [125]. Interestingly, HPD+VD increased expression of FGF23 in the kidney which correlated with an increase in circulating intact FGF23 but not c-terminal. FGF23 has previously been reported to be expressed in the kidney [126, 127] and intact FGF23 was increased by acute tubulointerstitial injury [128]. Although VD supplementation corrected serum Pi to NPD levels, the trabecular and cortical bone loss associated with HPD was not blunted. Further, it altered kidney gene expression consistent with early-stage calcification. A prior study found that male rats supplemented with vitamin D and calcium increased kidney stone formation and tubular calcifications [129]. The results do not support dietary VD supplementation as a viable strategy to counter the effects of a HPD on bone volume and may have negative consequences on the kidney.

Another strategy to understand the role of Pi-additives on Ca gut absorption is to increase dietary Ca in parallel with Pi. A study of prolonged high Pi intake in rats suggested that the increased intake of dietary Pi reduces Ca balance [68]. Increasing Ca and Pi has been shown to greatly reduce the HPD-induced increase in Pi-responsive serum factors FGF23, PTH, and OPN in male mice [28]. Results identified that Ca supplementation attenuated the systemic effects of HPD, including serum Pi, phosphatonins, and changes in kidney gene expression. However, Ca supplementation only partially rescued bone indices. In female mice, increased dietary Ca had no effect on HPD-induced reduction in trabecular or cortical bone volume however the HPD-increase in cortical porosity was blunted. Increased dietary Ca in male mice also had no effect on HPD-induced trabecular bone volume loss while cortical bone volume was rescued to near NPD levels. Interestingly, Ca did not blunt the HPD driven increase in cortical porosity. A study using a male rat model of polycystic kidney disease found that Ca supplementation increased trabecular bone volume and decreased cortical porosity suggesting that the ability of dietary Ca to improve various bone parameters may be at least partially influenced by the driver of bone loss [130]. The lack of a response of trabecular bone to increased dietary Ca might be explained by a recent study that identified a HPD-increase in inflammatory T cell populations in the bone marrow that was required for the decrease in trabecular bone volume [20]. The lack of a strong response to dietary Ca aligns with a study in twelve healthy female subjects found Ca supplementation with high Pi consumption corrected the HPD driven increase in PTH but not bone resorption markers [15]. A study analyzing the Korean National Health and Nutrition Examination Survey found a small benefit to bone with increasing Ca intake in males but not females and a slightly stronger benefit to a high Ca to Pi ratio [131]. Given that older adults often have low Ca intake and high Pi intake [47], promoting a high dietary Ca:Pi ratio may represent a simple lifestyle intervention to improve skeletal outcomes [19, 21, 23]. Particularly, as many current agents used to treat bone disease, such as osteoporosis, have side effects.

Among all therapeutics tested, only the bisphosphate, Zoledronic acid (Zol) which was injected ip, effectively preserved bone volume in mice fed HPD. Bisphosphonates are the most commonly used class of drugs to treat osteoporosis or metabolic bone disease [132]. These drugs act by reducing osteoclast activity and preserving bone and are currently administered annually by IV injection [133]. In HPD-fed mice, Zol did not alter serum chemistry or aspects of the Pi-homeostatic network (FGF23, PTH, or VD) relative to HPD alone, although, serum OPN levels increased relative to HPD. Zol completely inhibited the HPD-driven decrease in trabecular and cortical bone volume. These results are in agreement with a recent study using the same diets, which found a HPD-induced increase in osteoclast numbers and surface by histology of the tibia and reduced bone volume in HPD-fed mice and these were consequences were reversed by administration of a RANKL neutralizing antibody [20]. Taken together, the results identify osteoclastic resorption as required for the effects of HPD on bone.

Surprisingly, the current study found that although Zol blocked the HPD-driven loss of cortical bone in the femur, it exacerbated an HPD-driven increase in cortical porosity. Although bisphosphonates used to treat osteoporosis improve bone volume and are generally considered to decrease cortical porosity, an increase in cortical porosity in the context of Zol has been detected in studies under relatively specific instances, such as a CKD model in rats [120], teriparatide (human PTH [1–34]) treatment in ovariectomized rats [134], and aged ewes [135]. Published studies using Zoledronic acid in mice and rats generally range from 20 to 125 µg/kg [120, 136–140], with at least one study using 1000 µg/kg [141]. Relative to clinical use, the 20 µg/kg in mice is similar to a single dose of Zol (Reclast) for osteoporosis in humans whereas the 100 µg/kg dose is similar to what would be used in the context of bone metastasis in humans. Interestingly, in the context of CKD a 20 µg/kg dose generated an increase in cortical porosity however if that dose was divided into ten 2 µg/kg doses, cortical porosity was similar to control [120]. However, fractionated dosing of Risedronate, another bisphosphonate, in the same mouse model still showed increased cortical porosity [120]. Therefore, we cannot rule out the possibility that the increased porosity, in the context of HPD, may be due to the relatively high single dose of Zol. Alternatively, high PTH levels represent a commonality between the conditions (CKD, High Pi, and PTH treatment) that generate Zol-induced increased porosity leading to the speculation that this might represent a possible mechanism. In agreement, at least one studies in osteoporotic women identified an increase in porosity to PTH [1–34] treatment but no change to Zol alone [142]. Given the recent correlation between cortical porosity, aging [143, 144], and fracture risk [145–147] the results herein argue for the clinical monitoring of Pi consumption in patients recently started on Zol treatment.

Although Zol did not correct HPD-induced serum Pi or phosphatonin levels, it did blunt the HPD-driven decrease in expression of Klotho, VDR, and the Pi-transporters, while exacerbating the expression of inflammatory and macrophage associated genes in the kidney. Mechanistically, Zol acts inhibits farnesyl pyrophosphate (FPP) synthase, an enzyme of the mevalonate (cholesterol biosynthesis) pathway resulting in osteoclast apoptosis [148]. However, this pathway functions in other cell types and bisphosphonates have been shown to alter gene expression in other cell types including macrophages [149], T cells [150], and renal tubular epithelial cells [151]. An analysis of the Zol use using the US FDA’s Adverse Event Reporting System found that renal tubular necrosis was the most frequently reported adverse event for inpatients treated for breast cancer and osteoporosis [152]. It should be noted that a relatively high dose of Zol was used in this study and it cannot be ruled out that lower or fractionated doses might not generate such a response. Although speculative, the result suggests that the downregulation of Klotho, Pi transporters, and VDR in the kidney require the stimulation of the mevalonate pathway either directly or indirectly through neighboring cells. Further, these findings highlight a possible link between Zol’s anti-resorption effects and altered kidney gene expression, suggesting systemic communication between bone and kidney function.

Of clinical relevance, the Pi therapeutic strategies, VD, CNC, and Zol, triggered kidney gene expression changes indicative of immune activation or tissue injury when administered alongside HPD and normal kidney function. Dietary HPD+VD, stimulated expression of Pi homeostatic factors FGF23, CaSR, Cyp24a1, OPN, and inflammatory and injury genes CD11b, CD68, IL-17a, IL-6, IL-1β, and Kim-1 (Patterns 2 and 3). While the Pi homeostatic factors are known to be directly responsive to VD [153–155], the inflammatory factors are likely the results of dysregulated mineral homeostasis and possibly early-stage tissue dysfunction. Although osteoblast/osteocyte are considered the primary source of FGF23, certain disease and inflammatory conditions can induce expression in the kidney [126, 128, 156] and macrophages [157–159]. Mice fed HPD+CNC stimulated expression of mineral homeostatic factors and inflammatory genes but lacked expression of Cd11b and CD68 suggesting this may be a T and B cell response. Still, the physiological significance of the VD and CNC responses on kidney function remains to be determined. Finally, Zol stimulated an expression profile related to macrophage recruitment and inflammation. Consuming excess Pi additives is prevalent worldwide and these findings underscore the importance of evaluating mineral regulating therapies for the potential side effects in the context of a high Pi diet.

High dietary Pi consumption alone did not produce a significant increase in mineral deposition in the kidney after 5wks, although some genes associated with regulation of calcification, inflammation, and damage such as osteopontin, Lcn2, and Kim1 respectively, were detected as elevated. After 10wks on the high Pi diet a significant increase mineral was detected. This corresponded with increased expression of the above genes and additionally macrophage genes Cd11b and CD68. This is agreement with a study in healthy mice fed a high Pi diet that identified through histology, the recruitment of macrophages to injured proximal tubular cells starting around 2 months [78]. Although the mechanism(s) was not investigated herein, recent studies provide a potential explanation. One mechanism by which high Pi intake might increase inflammation and mineralization in the kidney is through an increase in oxidative stress. Hyperphosphatemia has been linked to oxidative stress which is also tightly linked to inflammation [160–163] and calcium deposits in the kidney [164, 165].

## Conclusions

In summary, the study advances the understanding of the health impacts of Pi additives which are common in ultra-processed foods and common in the Western style diet. Specifically, the results produce translationally relevant findings that simply controlling serum Pi and/or Ca levels or Pi responsive endocrine factors such as FGF23, PTH, and osteopontin with existing clinically used mineral homeostasis therapies is not sufficient to block high dietary Pi induced reduction in bone volume, although some offer relief from high Pi diet increased cortical porosity. Further, this study provides new insight into how clinically relevant nutritional and therapeutic strategies used to normalize mineral homeostasis affect bone and kidney in the context of normal kidney function. Mechanistically, this study provides new knowledge related to interconnected complex systemic endocrine network responsible for regulating serum Pi and Ca and its long-term effects on tissue function.

## Electronic supplementary material

Below is the link to the electronic supplementary material.

Supplementary Material 1

## Data Availability

The datasets used and analyzed during the current study are available from the corresponding author upon reasonable request.

## Abbreviations

Pi: Phosphorus/Inorganic phosphate
Ca: Calcium
LPD: Low Phosphate Diet
NPD: Normal Phosphate Diet
HPD: High Phosphate Diet
LCPD: Low Calcium and Phosphate Diet
HCPD: High Calcium and Phosphate Diet
BUN: Blood Urea Nitrogen
ALP: Alkaline Phosphatase
VD: Vitamin D
LaC: Lanthanum Carbonate
CNC: Cinacalcet
Zol: Zoledronic acid
FGF23: Fibroblast Growth Factor 23
PTH: Parathyroid Hormone
OPN: Osteopontin
µCT: Micro-Computed Tomography
CTX: Cross-linked C-telopeptide of type I collagen
P1NP: Procollagen type-1 intact N-terminal Propeptide
BV/TV: Bone Volume/Tissue Volume
Tb.N: Trabecular Number
Tb.Th: Trabecular Thickness
Tb.Sp: Trabecular Spacing
Ct.Ar: Cortical Area
Ct.Th: Cortical Thickness
Ct.Po: Cortical Porosity
Po.V: Pore Volume
CKD: Chronic Kidney Disease
